# Longer-term increased cortisol levels in young people with mental health problems

**DOI:** 10.1016/j.psychres.2015.12.025

**Published:** 2016-02-28

**Authors:** Kareen Heinze, Ashleigh Lin, Renate L.E.P. Reniers, Stephen J. Wood

**Affiliations:** a School of Psychology, University of Birmingham, UK; b School of Psychology, Keele University, UK; c Telethon Kids Institute, The University of Western Australia, Australia; d Melbourne Neuropsychiatry Centre, Department of Psychiatry, University of Melbourne and Melbourne Health, Carlton South, VIC, Australia

**Keywords:** Hair cortisol, Youth mental health, Clinical staging

## Abstract

Disturbance of hypothalamus–pituitary–adrenal axis activity is commonly reported in a range of mental disorders in blood, saliva and urine samples. This study aimed to look at longer-term cortisol levels and their association with clinical symptoms. Hair strands of 30 young people (16–25 years) presenting with mental health problems (*M*_age_±SD=21±2.4, 26 females) and 28 healthy controls (HC, *M*_age_±SD=20±2.9, 26 females) were analyzed for cortisol concentrations, representing the past 6 months prior to hair sampling. Clinical participants completed an assessment on psychiatric symptoms, functioning and lifestyle factors. All participants completed the Perceived Stress Scale. Hair cortisol concentrations representing the past 3 (but not 3–6) months were significantly increased in clinical participants compared to HC. Perceived stress in the past month was significantly higher in clinical participants compared to HC, but not significantly correlated with hair cortisol. Hair cortisol levels were not significantly associated with any other measures. Hair segment analyses revealed longer-term increased levels of cortisol in the past 3 months in early mental health problems. Further insight into the role of cortisol on the pathogenesis of mental illnesses requires longitudinal studies relating cortisol to psychopathology and progression of illness.

## Highlights

• Investigation of hair cortisol in young people with mental health problems.
• Elevated first segment cortisol in clinical participants compared to controls.
• Hair cortisol was not associated with clinical measures.

## 1 Introduction

Disturbances in hypothalamus–pituitary–adrenal (HPA) axis diurnal activity and responsivity are common findings in a range of mental disorders (e.g. Yehuda et al., 1993; Meewisse et al., 2007; Knorr et al., 2010). One of the most commonly reported parameters of the HPA axis is the glucocorticoid hormone cortisol. Over- and under-activity of cortisol concentrations have been reported by means of blood, saliva and urine samples in mood disorder (Cervantes et al., 2001, Vreeburg et al., 2009), psychosis (Ryan et al., 2004), posttraumatic stress disorder (PTSD) (Yehuda et al., 1990), panic (Bandelow et al., 2000) and generalized anxiety disorder (GAD) (Mantella et al., 2008), somatization syndrome (Rief et al., 1998), and eating (Monteleone et al., 2001) and substance use (Adinoff et al., 2003) disorders. Despite this, there is considerable variability across (Yehuda et al., 1993) and within diagnostic categories (for meta-analyses in PTSD, see Meewisse et al. (2007), and depression, see Knorr et al. (2010)), which is why it is important to investigate whether cortisol fluctuations can be explained by factors other than specific diagnostic categories, such as inter-individual differences and stressor characteristics (Miller et al., 2007).

Established analyses of cortisol in *saliva*, *plasma* and *urine* have proven to be useful and reliable tools for documenting *real-time* circulating cortisol levels (*plasma*, *saliva*) or mean cortisol excretions over a specific time, usually 24 h (*urine*). In contrast, *hair* cortisol represents a reliable, *longer-term* measure (generally up to months) of stress and endogenous cortisol concentrations (Stalder and Kirschbaum, 2012). The advantages of hair cortisol assessment lie in providing a retrospective examination of hair cortisol levels over an extended time period that is virtually impossible to achieve with other methods. Moreover, it is a non-invasive sampling method, samples can be easily stored at room temperature for an extended period, and sampling avoids problems of adherence which often experienced with other methods. However, hair cortisol concentrations decrease from more proximal to distal segments in human scalp hair, limiting the retrospective period of examination (Stalder and Kirschbaum, 2012).

Considering a generally accepted human scalp hair growth rate of 1 cm per month (Wennig, 2000) and taking hair samples from the scalp near the posterior vertex region, this method allows for retrospective capture of cortisol concentrations for up to six months (Dettenborn et al., 2012). Recent studies in clinical populations have demonstrated increased hair cortisol concentrations in depression (Dettenborn et al., 2012), PTSD (Steudte et al., 2011a), and in alcohol-dependent individuals (Stalder et al., 2010), and decreases in GAD (Steudte et al., 2011b), and PTSD (Steudte et al., 2013). Occupational impairment (i.e. unemployment) has also been shown to be associated with increased hair cortisol (Dettenborn et al., 2010). Strong test–retest associations for repeated hair cortisol measurements have been revealed, indicating high intra-individual stability. Structural equation modelling showed that, if no major life events or other stressors are present, hair cortisol assessments comprise a strong trait component which explains between 59% and 82% of variance in cortisol levels (Stalder et al., 2012). A recent systematic review of hair analyses revealed variations in hair cortisol (Staufenbiel et al., 2013) that are similar to findings from meta-analyses and reviews on more established cortisol measures (Meewisse et al., 2007, Knorr et al., 2010). Despite some inconsistencies, hair cortisol appears to be increased in depression, and decreased in anxiety disorders (Staufenbiel et al., 2013).

The period of adolescence and young adulthood is characterized by increased vulnerability for the development of mental disorders. Half of the lifetime cases of mental disorder start by age 14 and three quarters by age 24 (Kessler et al., 2005). It is hypothesized that adolescence is accompanied by a biological sensitivity to stress and that age-related cortisol increase may trigger the expression of symptoms in vulnerable individuals (Walker et al., 2010). Higher cortisol levels predict a higher risk of conversion to psychotic disorder in at-risk individuals, providing support for this hypothesis (Walker et al., 2010). The pathogenesis of childhood anxiety disorders appears similar: high levels of cortisol may induce changes to subcortical circuits, which may make children vulnerable to developing anxiety symptoms (Muris, 2006). In one study, Rao et al. (2008) exposed adolescents with depressive disorder and healthy controls (HC) to psychosocial stress. Both groups showed increased cortisol levels, but individuals with depression displayed an increased and sustained cortisol response. This supports the notion that stressful events play a role in the development and maintenance of depressive symptoms (Rao et al., 2008).

The time between the onset of the disorder (or stressful or traumatic event) and hair collection appears to be a crucial element in explaining the diversity of cortisol findings. Luo et al. (2012) reported increased hair cortisol one month after a traumatic event in adolescents with PTSD, with levels decreasing after 7 months (for review, see Staufenbiel et al. (2013)). Increased cortisol levels are therefore likely to reflect the on-going stress and not the disorder itself (Staufenbiel et al., 2013). A significant difference was observed between early- and late-onset bipolar disorder. Patients with a late onset disorder (≥30 years) presented with higher hair cortisol than the early-onset group and HC (Manenschijn et al., 2012). This finding suggests that early onset bipolar disorder may be more strongly linked to a genetic vulnerability while late onset is usually triggered by life events and stress (Staufenbiel et al., 2013). Taken together, these findings suggest that HPA axis activity is elevated at the time of stressor onset with declining cortisol levels as time passes (Miller et al., 2007). However, the exact timeline of these hormonal changes is unclear; that is, how long does the hypothesized cortisol increase persist?

An alternative to explaining the heterogeneity of HPA axis findings by classifying mental ill health into disorder-specific categories is to use a dimensional approach. In this way, one can examine symptom severity and consider a timeline of an individual's symptoms. One example of a dimensional approach is a clinical staging model. Within this framework, mental disorders are assumed to develop from a *pluripotential state*, consisting of undifferentiated general symptoms (such as depressive and anxiety symptoms), and from a background of specific and non-specific risk factors (McGorry et al., 2006, Lin et al., 2013), which are associated with non-specific *distress* (McGorry, 2013). This idea is supported by the recent finding of a common mental distress factor underlying depressive, anxiety, and psychotic phenomena in adolescents (Stochl et al., 2015). It can therefore be inferred from the clinical staging model that the early stages of mixed mental health problems are likely to coincide with elevated cortisol levels. However, to our knowledge, there has been no study investigating longer-term cortisol levels in youth with early mental health problems from a clinical staging perspective.

The aim of this study was to investigate the pathogenesis of mental disorders in adolescents and young adults, and whether diverse psychiatric symptoms are associated with altered longer-term cortisol levels. We included young people who had sought help for mental health problems, as well as HC. We wanted to test the hypothesis that early stages of mental health problems are associated with significant distress (McGorry, 2013) and therefore elevated cortisol levels. We further explored the association between cortisol levels and general psychological distress, depressive, anxiety and psychotic symptoms, alcohol and tobacco use, and childhood traumatic experiences.

## 2 Methods

### 2.1 Participants

Thirty-one participants seeking help for mental health problems were recruited from the South Birmingham area via clinical services (Youthspace & Birmingham Healthy Minds). Inclusion criteria were being aged 16–25 years and recently help-seeking (within 6 months of clinical contact) for mental health problems. Youthspace is a youth-focused secondary mental health service that provides support for youth aged 16–25 years. The service sees young people with a variety of diagnoses and has no specific exclusion criteria. Youthspace offer a variety of treatments and case management is provided by a multi-disciplinary team. Birmingham Healthy Minds is a primary care psychological therapy service, offering brief psychological talking therapy for individuals aged 16 and above who present with depressive and anxiety symptoms. Their exclusion criteria are bipolar disorder, psychosis, suicidality or need for long-term care. At the time of recruitment, both services operated primarily though GP referral. All clinical participants from both services were generally eligible for the study. Participants were recruited in one of three ways: (1) via their clinician; (2) approached by the researcher in the waiting room before or after their appointment at Youthspace; or (3) by responding to a poster advertisement at these services. Not all eligible individuals at the services were approached for participation. Reasons for this are because their clinicians may not have felt that participation was appropriate (e.g. they were in crisis), or because a researcher was not at the clinic on the days of their appointments. Both services operated via multiple clinicians at multiple sites, and therefore due to practical reasons, it was not possible to gather statistics on refusal rates. However, not all service users who were initially approached took part in the study. Obtaining consent to partake meant in the first instance, taking part in an interview and self-report study focusing on mental health symptoms, functioning and general life style factors. In the second instance, individuals who either had a family history with mental health problems in a first-degree relative or who subjectively did not improve concerning their well-being after six weeks of this interview and self-report study, were asked to donate a hair sample.

Twenty-eight HC were recruited via university staff and local advertisements at the University of Birmingham, United Kingdom. HC were age, gender, occupation and education matched to the clinical participants, and had no personal or first-degree family history of mental illness. A personal history was excluded based on the Structured Clinical Interview for DSM-IV-TR (American Psychiatric Association, 2000) Axis I Disorders.

Exclusion criteria for both groups were hair length of less than 3 cm at the posterior vertex region, a lack of sufficient English or cognitive ability to provide informed consent and adequately complete assessments. The study was approved by the local research ethics committee and participants provided written informed consent.

### 2.2 Hair cortisol analyses

Hair strands of approximately 3 mm diameter were taken from a posterior vertex position, as close to the scalp as possible. Cortisol concentrations were determined from the 3 cm hair segment most proximal to the scalp, and the following 3 cm segment in accordance with the protocol of Stalder et al. (2012). Segments were gently mixed with 2.5 ml isopropanol for three minutes. After drying, 7.5 mg of whole, non-pulverized hair was incubated in 1800 µl methanol for 18 h at 45 °C. Cortisol levels were determined using a commercially available immunoassay with chemiluminescence detection (CLIA, IBL-Hamburg, Germany).

### 2.3 Measures

Information on regular medication intake was collected and covered exposure to contraceptive hormones (oral intake, patches or implants) or glucocorticoids, and psychiatric medication (antidepressants, neuroleptics, betablockers, anticonvulsants) in the past 6 months. All participants self-reported demographic and hair-related information (number of hair washes per week, hair treatments) and completed the 10-item Perceived Stress Scale (PSS) (Cohen et al., 1983). The PSS evaluates the extent to which situations in one's life are appraised as stressful and the degree to which a person rates their life as unpredictable, uncontrollable and overloading (Cohen et al., 1983). The clinical group additionally completed an interview and self-report assessment on the following measures:

#### 2.3.1 Comprehensive Assessment of At-Risk Mental States (CAARMS)

The CAARMS (Yung et al., 2005) is a semi-structured interview designed to determine the at-risk for psychosis mental state. The four subscales are Unusual Thought Content (e.g. delusional mood, overvalued ideas), Non-Bizarre Ideas (e.g. suspiciousness, grandiosity), Perceptual Abnormalities (e.g. distortions, illusions, hallucinations), Disorganized Speech (e.g. difficulties with speech and communication) are rated on severity (0=absent/never, 6=psychotic and severe) and frequency (0=absent/never, 6=continuous) of psychotic symptoms. Severity and frequency were summed to produce a total score across the four subscales. Good to excellent agreement for intra-class correlation coefficients were reported for CAARMS subscales with an overall score for inter-rater reliability of 0.85. CAARMS criteria displayed good concurrent (e.g. with the Brief Psychotic Rating Scale) and predictive validity (e.g. higher risk of transition to psychosis in individuals with an at-risk mental state) (Yung et al., 2005).

#### 2.3.2 Kessler Psychological Distress Scale (K-10)

The K-10 (Kessler et al., 2002) is a 10-item questionnaire assessing psychological distress via questions about depressive and anxiety symptoms in the past 30 days. Items are rated on a 5-point scale with total scores ranging from 10 to 50. The K-10 is a moderately reliable instrument (kappa ranging from 0.42 to 0.74) (Dal Grande et al., 2002) and demonstrates good concurrent validity with other mental health instruments such as the General Health Questionnaire and current diagnosis of anxiety and affective disorders (Andrews and Slade, 2001).

#### 2.3.3 Quick Inventory of Depressive Symptoms (QIDS)

The QIDS (Rush et al., 2003) is a 16-item, semi-structured interview to gauge severity of depressive symptoms over the past seven days. Items are scored 0–3 and total scores range from 0 to 27. A meta-analysis demonstrated acceptable psychometric properties with internal consistencies ranging from 0.65 to 0.87 (Chronbach's *α*) and concurrent validity (Reilly et al., 2015).

#### 2.3.4 Ruminative style

Ruminative style was measured on a 10-item scale, requiring participants to indicate on a 4-point scale how often they engage with certain thoughts when they are feeling down or depressed (Leach et al., 2008). Total scores range from 10 to 40.

#### 2.3.5 Childhood Trauma Questionnaire, short form (CTQ-SF)

The CTQ-SF (Bernstein et al., 2003) is a 28-item instrument generating information on traumatic childhood experiences using a 5-point scale. Subscales are sexual, physical and emotional abuse, and emotional and physical neglect, with subscale scores from 5 to 25, and a total score from 25 to 125. The CTQ demonstrates good internal consistencies with Chronbach's *α* ranging from 0.79 to 0.94, and high retest-reliability over 6 months with an intra-class correlation of 0.88, as well as concurrent validity with the full CTQ (Bernstein et al., 1994, 2003).

#### 2.3.6 Overall Anxiety Severity and Impairment Scale (OASIS)

The OASIS (Campbell-Sills et al., 2009) is a brief 5-item questionnaire of severity and impairment across multiple anxiety disorders and subthreshold anxiety. It captures frequency and intensity of anxiety, avoidance behaviour and interference of anxiety with everyday life and relationships. Total scores range from 0 to 20. The OASIS showed convergence with major anxiety measures, a Chronbach's *α* of 0.84 for the five items (Campbell-Sills et al., 2009), and one-month retest reliability of 0.82 (Norman et al., 2006).

#### 2.3.7 Psychosocial functioning

The Global Functioning: Social (Auther et al., 2006) and Role (Niendam et al., 2006) Scales were used to index current social and role functioning, providing overall scores from 1 to 10, with 10 indicating superior functioning and 1 extreme dysfunction. Inter-rater reliability for social and role functioning ranged from 0.85 to 0.95, and the social functioning scale was significantly correlated with social contacts (*r*=0.70) and role functioning with work and school functioning (*r*=0.57), demonstrating construct validity (Cornblatt et al., 2007).

#### 2.3.8 Lifestyle factors

Body mass index (BMI) was assessed. The frequency of smoking and alcohol consumption in the past three months, and a total score for tobacco and alcohol acuity as measured by the Alcohol, Smoking and Substance Involvement Screening Test (ASSIST) (Humeniuk et al., 2008) were provided. The ASSIST is a brief screening questionnaire for hazardous, harmful and dependent use of alcohol, tobacco and other psychoactive substances (cannabis, cocaine, amphetamines, sedatives, hallucinogens, inhalants, opioids and other non-specified drugs). It provides information about substances people may have ever used, used in the past three months, problems related to peoples' substance use, risk of current and future harm and dependence. Scores ranging from 0 to 39 can be classified into *low* (for alcohol 0–10, all other substances 0–3), *moderate* (alcohol 11–26, other substances 4–26) and *high* (all 27+) substance acuity (Henry-Edwards et al., 2003). The calculation of Cronbach's *α* revealed good inter-item correlation for the individual scales ranging from 0.77 to 0.94. Concurrent validity has been shown with the Addiction Severity Index with correlations for scale scores ranging from 0.76 to 0.88 (Humeniuk et al., 2008). No clinical participant reached a total score for any psychoactive substance (other than tobacco and alcohol) in the range of moderate acuity or above (except moderate acuity for six clinical participants for cannabis, and for two participants for sedatives), which did not allow for adequate statistical testing due to small sample sizes.

### 2.4 Statistical analyses

Hair samples from 31 clinical participants and 28 HC were collected. We initially approached 57 clinical participants (from those who took part in the interview and self-report study) and 35 HC to donate hair samples. Five male and one female clinical participant, and four male and three female HC could not participate due to too short or too little hair. Whereas those males were predominantly white-British, all four females were either mixed-race or black-African. Fourteen of the other 21 participants initially approached were either no longer contactable or did not want to participate, two moved away after being approached, one was too busy, and four did not meet criteria for another joint study (which is why they were not asked to partake), resulting in an overall refusal rate of 47%. Data of the first hair segment of one clinical participant was excluded due to an extreme outlier (>90 standard deviations above the mean), providing data for 30 clinical participants and 28 HC in the first hair segment, and due to short hair, data for 24 clinical participants and 24 HC on the second hair segment. Cortisol data was positively skewed (skewness first segment=2.6, skewness second segment=3.73, kurtosis first segment=7.65, kurtosis second segment=19.24), but analyzed using robust *t*-tests and analyses of covariance (ANCOVA).

Independent samples *t*-tests were performed for group comparisons of cortisol, age and PSS scores between clinical participants and HC, and between clinical participants taking antidepressants and those who were not, to discern whether antidepressant treatment could be associated with alterations in cortisol concentrations. For categorical data, *χ*^2^-tests were used to evaluate group differences. We conducted Spearman correlations to discern associations of cortisol levels with clinical measures and lifestyle factors in the clinical group, and for associations between the first and second hair segment. A paired samples *t*-test was conducted to compare mean hair cortisol levels in the first and second segment, as well as a repeated measured analysis of variance (ANOVA) to illustrate the interaction of cortisol levels over time. ANCOVA were employed to compare clinical participants and HC, with ethnicity as a covariate, since groups differed significantly on ethnicity and it can be a confounding variable (Wennig, 2000, Wosu et al., 2015).

## 3 Results

### 3.1 Demographics and clinical description

Clinical participants and HC did not differ significantly in terms of age, gender, years of education and occupation, but differed significantly with regards to ethnicity (see Table 1). Median time between gathering state-specific clinical measures (QIDS, CAARMS, K-10, OASIS, functioning, smoking, alcohol consumption, BMI) and hair collection/gathering hair-related variables (PSS-scores and medication intake, and demographics for HC) was 18.5 days (range 0–71 days). State-specific measures, along with data on ruminative style, and childhood trauma are presented in Table 2.

Twenty three clinical participants described their major mental health problem as depression, four as bipolar disorder, 13 as anxiety, two as eating disorders, and one as obsessive-compulsive disorder. Ten were classified as ultra-high risk for psychosis, 20 reported to have self-harmed and 18 had attempted suicide.

### 3.2 Hair cortisol and perceived stress: clinical participants vs HC

**Fig. 1:**
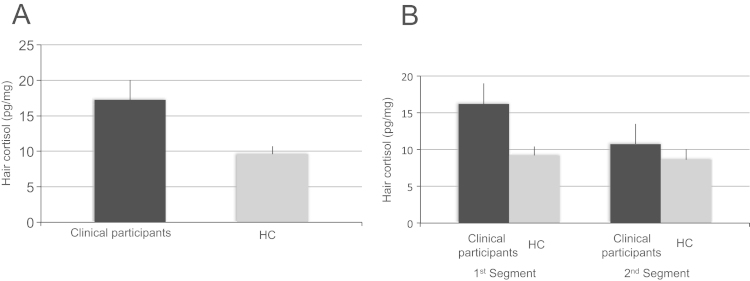
(A) Hair cortisol concentrations (1st segment) in clinical participants with mental health problems (*n*=30) compared to healthy controls (HC; *n*=28, *p*=0.016); (B) decrease in hair cortisol in clinical participants and HC from first (*n_Clinical_*=24; *n_HC_*=24) to second hair segment (*n_Clinical_*=24; *n_HC_*=24).

There was a significant elevation of cortisol concentrations for clinical participants compared to HC for the first hair segment, representing the past three months prior to sampling (*t* (56)=2.489, *p*=0.016; *d*=0.66) (see Fig. 1A). There were no group differences in cortisol levels for the second segment representing the past three to six months prior to sampling (*p*=0.495; *d*=0.2). Repeated analyses without individuals taking glucocorticoids did not change the findings for the first (*t*(54)=2.501, *p*=0.015) and second segment (*p*=0.473). Clinical participants perceived significantly more stress in the past month compared to HC (*t*(56)=11.202, *p*<0.001) (see Table 1); however, there were no statistically significant correlations between cortisol concentrations in the first segment and perceived stress, for the whole sample (*r*=0.219, *p*=0.099) or groups compared individually (*r*_*Clinical*_=0.144, *p*=0.448; *r*_*HC*_=−0.201, *p*=0.305). Hair cortisol of the first and second hair segment correlated significantly with each other (*r*_*48*_=0.286, *p*=0.049). A paired-samples *t*-test did not reveal a significant decrease of cortisol over time (*t* (47)=1.601, *p*=0.116). There was no significant Group×Time interaction of cortisol levels (*F* (1, 46)=1.704. *p*=0.20; see Fig. 1B). The main finding of elevated cortisol concentrations in the first hair segment in clinical participants compared to HC remained significant after controlling for ethnicity (*F* (1, 55)=4.77, *p*=0.012).

### 3.3 Hair cortisol, clinical measures, functioning, and lifestyle

Hair cortisol concentrations in the first and second segment were not significantly correlated with any of the investigated clinical measures, social and role functioning or lifestyle measures (all *p*>0.05).

### 3.4 Hair cortisol and medication

Both groups were matched in terms of glucocorticoid and contraceptive hormone use, making this unlikely to account for group differences in cortisol in the first hair segment: One clinical participant and one HC were using glucocorticoids at the time of hair collection, of which neither produced abnormally elevated cortisol levels in either hair segment. 24% of the clinical participants and 36% of HC were exposed to contraceptive hormones at the time of hair collection.

72% of the clinical participants were taking antidepressants, 3.4% neuroleptics, 3.4% anti-convulsive medication, and 3.4% were taking beta-blockers at the time of hair collection. There were no significant differences for cortisol levels in the first (1st) and second hair (2nd) segment in those clinical participants taking antidepressants and/or other psychiatric medication (*n*_*1st*_=21; *n*_*2nd*_=16) compared to those patients who were not (*n*_*1st*_=9; *n*_*2nd*_=8) (*p*>0.05).

## 4 Discussion

In the present study, we identified elevated hair cortisol concentrations representing the past three months prior to hair sampling in adolescents and young adults with mental health problems compared to healthy participants. We also found elevated perceived stress in clinical participants, but there was no association between cortisol and subjective stress experience. There were no significant correlations between cortisol and clinical measures, functioning and lifestyle factors.

Our finding of longer-term elevated cortisol levels in young individuals with mental health problems is in line with the presumption of the experience of non-specific but significant psychological distress in the early stages of mental health problems (McGorry, 2013), and therefore with a dimensional approach to emerging mental health disorders such as a clinical staging model (McGorry et al., 2006, Lin et al., 2013). We confirmed that this state of undifferentiated symptoms in the early stages of a mental health disorder is associated with an increase in cortisol and self-reported stress levels. However, whether decreases in cortisol levels coincide with illness progression (dependent or independent of diagnostic category), and whether cortisol levels decline (to normal) with remission of symptoms, remains to be investigated longitudinally.

Our clinical participants were experiencing mental health problems for at least six months, although had only recently sought help. The time when the young people started seeking help can be seen as a *crisis point*, when their problems started to worsen (possibly due to a significant event or stressor), explaining elevated cortisol only in the past three months. Although there was no significant time-by-group interaction, visual inspection of the data suggest a much more pronounced increase in cortisol level for the clinical group. This non-significance may be due to washout effects (Kirschbaum et al., 2009) and variable, inter-individual rates of hair growth (Wennig, 2000) that may limit the detection of differences in the more distal hair segment. This null finding may also have been due to insufficient statistical power.

In most previous studies, individuals with full-blown mental disorders (usually at the chronic stage) have been investigated, whereas the current sample included help-seeking youth in the early stages of mental health problems. Specific patterns of hair cortisol alterations were reported in previous studies, e.g. increases in depression (Dettenborn et al., 2012), and (if diagnosis sustained at least in the medium-term) decreases in PTSD (Steudte et al., 2013). However, we did not note correlations of cortisol with specific clinical measures (e.g. depressive, anxiety or psychotic symptoms). Given the relatively small sample size, but highly heterogeneous and comorbid sample in terms of clinical symptoms, it is conceivable that the results were masked by the interaction of symptoms with each other. For example, traumatic experiences in childhood might result in reduced cortisol levels while depressive symptoms result in increased cortisol, thus cancelling each other out. Furthermore, previous studies included clinical participants with more severe symptoms than in our sample; the more narrow range in the current study may have prevented the detection of correlations with cortisol.

The lack of correlations between hair cortisol and perceived stress in this study, is not an uncommon finding (Hjortskov et al., 2004, Stalder and Kirschbaum, 2012, Staufenbiel et al., 2013). Alternative explanations for this lack of association between cortisol and the PSS are the mismatch of time frame of one month for the PSS and 3 months for the first hair segment, or possibly the lack of validity of perceived stress in increasing hair cortisol concentrations (Staufenbiel et al., 2013). It is plausible that only certain characteristics of stressors lead to an endocrine response, which may have not been reflected using the PSS (Hjortskov et al., 2004).

It has been shown that hair cortisol alterations are implicated in a variety of health conditions (Staufenbiel et al., 2013), which is consistent with the finding of elevated cortisol in our sample of individuals with a range of mental health symptoms and disorders. Even though there is some evidence that cortisol might contribute to the aetiology of psychiatric conditions such as depressive symptoms (Johnson et al., 2006), the most plausible explanation appears to be a reciprocal relationship between stress promoting the development of mental disorders and psychiatric conditions being naturally accompanied by distress and cortisol increase.

### 4.1 Limitations

A limitation of this study is that clinical measures were not administered at the same time as the hair sample, although we aimed to collect clinical and cortisol data as close together as possible. A possible explanation for the lack of association between cortisol and perceived stress and other measures is that they did not entirely correspond with the window of cortisol detection. Although the median time from clinical assessment to cortisol measurement was only 18.5 days, cortisol measurements corresponded approximately with the past three and three to six months, whereas some self-reported and interview measures captured information from the previous week or month only. The HC group did not receive clinical assessments, therefore we were not able to use these clinical variables, functioning and lifestyle factors as covariates in our analyses. HC did not present with any threshold DSM disorder, however, we cannot fully rule out the influence of sub-threshold distress or disorders in this group.

In terms of feasibility and refusal rates of this study is important to mention that more males and more mixed-race women did not participate. Our clinical group included significantly more white individuals than the control group, whereas controls included more non-white (e.g. Asian and mixed-race) participants. Wosu et al. (2015) found differences in hair cortisol levels according to ethnicity, with lower levels in white participants. However, since our clinical group included more white participants, ethnicity is unlikely to account for our main finding of elevated hair cortisol. It is further unlikely that increased cortisol levels in the clinical group were due to antidepressant intake, as there were no differences in hair cortisol between those clinical participants who were taking antidepressant medication and those who were not. Furthermore, we were not able to obtain statistics on the representativeness of our clinical population; that is, what proportion of service users decided to take part in the study. Also, this study is only representative for young help-seeking people who either have a family history of mental health problems or whose problems are sustained for at least 6 weeks.

A relatively small sample size is a further limitation, although we found, as hypothesized, a group difference in hair cortisol concentration in the first segment. Given the clinical heterogeneity of the sample, the sample size was not sufficient to conduct subgroup analyses (e.g. comparing different types of anxiety disorders, or clinical stages). Differentiation of specific mental disorders on the basis of increases or decreases in cortisol [as seen in previous studies, e.g. for review, see Staufenbiel et al. (2013)] is more likely to occur where there is persistence and progression of symptoms, and therefore more likely to be distinguished over the future course of illness in this population.

Lastly, we cannot rule out the influence of recreational tobacco use, alcohol consumption, and use of psychoactive illegal drugs on hair cortisol levels. We did not find an association between hair cortisol and alcohol and tobacco use in the past three months, nor an association with tobacco- and alcohol-related problems or behaviours. Parrott et al. (2014) found an almost 4-fold increase in hair cortisol levels in regular ecstasy users as compared to non-users. However, the sample size was too small to conduct meaningful analyses with any illegal psychoactive drugs.

### 4.2 Conclusions and future directions

We found elevated hair cortisol levels in young people with diverse mental health problems, which is consistent with the finding of increased HPA axis activity with stressor onset, or in our case, in the early stages of help-seeking for mental health problems. Future research should focus on disentangling how actual life stressors and subjective stress experience are associated with increases in cortisol and the development and maintenance of mental disorders, and how HPA axis activity develops and adapts over the course of illness and with remission of symptoms.

## Financial disclosures

This study was partially financially supported by the Pump Priming Research Grant of the Wellcome Trust Institutional Strategic Support Fund. Ashleigh Lin is supported by the NHMRC (#1072593). Renate Reniers is funded by an MRC Research Grant (#MR/K013599/1).

## Figures and Tables

**Table 1:** Demographic and hair-related information on clinical participants and HC.

	**Clinical participants (*****n*****=30)**	**HC (*****n*****=28)**	**Test statistic**	***p*****-Value**
*Demographics*				
Age (*M*±SD) in years	21±2.4	20±2.9	*t* (56)=1.32	0.19
Gender (*m*/*f*)	4 (13%)/26 (87%)	2 (7%)/26 (93%)	*χ*^2^(1)=0.6	0.44
*Ethnicity*				
White	26 (87%)	16 (57%)		
Asian	1 (3%)	5 (18%)	*χ*^2^(3)=8.9	0.03⁎
Black	2 (7%)	1 (4%)		
Mixed-race	1 (3%)	6 (21%)		
*Occupation*				
University student^a^	9 (30%)	9 (32%)		
College/A-Levels	9 (30%)	8 (29%)	*χ*^2^(3)=3.92	0.27
Unemployed	7 (23%)	2 (7%)		
Employed^b^	5 (17%)	9 (32%)		
*Years of education*				
>13	5 (17%)	7 (25%)		
13	15 (50%)	14 (50%)	*χ*^2^(2)=0.83	0.66
<13	10 (33%)	7 (25%)		
Hair-related variables				
* Washes per week* (*x* ˜)	3.5	3.5	*U*=372.5, *Z*=−0.52	0.6
* Hair treatment*^c^ (%)	63.3	42.9	*χ*^2^(1)=2.44	0.12
				
Stress questionnaire				
PSS score, *M±SD*	26.1±4.5	12±5.1	*t* (56)=11.20	<0.001⁎⁎⁎

*Notes.* HC=healthy controls, PSS=Perceived Stress Scale.

a Undergraduate and postgraduate university students.

b Working full or part-time, volunteering, and non-paid internships.

c Hair treatment in the past 6 months (hair coloration, dye, and perm).

⁎ *p*<0.05.

⁎⁎⁎ *p*<0.001.

**Table 2:** Clinical measure, functioning scores and lifestyle factors of clinical participants.

**Measure**	***M*±SD or Median**	**Range**
QIDS (*n*=30)	10.3± 4	4–20
Ruminative style (*n*=29)	30.7±5.2	19–40
CAARMS (*n*=30)	14.3±10	0–30
K-10 (*n*=30)	29.7±7.0	19–43
OASIS (*n*=30)	8.9±4.6	0–17
CTQ-SF (*n*=25)	50.4±18.2	27–83
ASSIST alcohol (*n*=30)	6.9±5.5	0–19
ASSIST smoking (*n*=30)	3	0–31
BMI (*n*=28)	22.7	16.9–36.4
Smoking frequency^a^ (*n*=30)	“Monthly”	“Never”–“daily”
Alcohol consumption^a^ (*n*=30)	“Weekly”	“Never”–“daily”

*Notes*. QIDS=Quick Inventory of Depressive Symptoms, CAARMS=Comprehensive Assessment of At-Risk Mental States, K10=Kessler Psychological Distress Scale, OASIS=Overall Anxiety Severity and Impairment Scale, ASSIST=Alcohol, Smoking and Substance Involvement Screening Test, BMI=Body Mass Index, *M*=mean score, SD=Standard deviation.

a Median scores for smoking and alcohol consumption frequency in the past three months are calculated based on self-report data ranging from 0 (never), 2 (once or twice), 3 (monthly), 4 (weekly), and 6 (daily or almost daily).
